# The Use of Partial Least Square Regression and Spectral Data in UV-Visible Region for Quantification of Adulteration in Indonesian Palm Civet Coffee

**DOI:** 10.1155/2017/6274178

**Published:** 2017-08-20

**Authors:** Diding Suhandy, Meinilwita Yulia

**Affiliations:** ^1^Laboratory of Bioprocess and Postharvest Engineering, Department of Agricultural Engineering, The University of Lampung, Jl. Soemantri Brojonegoro No. 1, Gedong Meneng, Bandar Lampung, Lampung 35145, Indonesia; ^2^Department of Agricultural Technology, Lampung State Polytechnic, Jl. Soekarno Hatta No. 10, Rajabasa, Bandar Lampung, Lampung, Indonesia

## Abstract

Asian palm civet coffee or kopi luwak (Indonesian words for coffee and palm civet) is well known as the world's priciest and rarest coffee. To protect the authenticity of luwak coffee and protect consumer from luwak coffee adulteration, it is very important to develop a robust and simple method for determining the adulteration of luwak coffee. In this research, the use of UV-Visible spectra combined with PLSR was evaluated to establish rapid and simple methods for quantification of adulteration in luwak-arabica coffee blend. Several preprocessing methods were tested and the results show that most of the preprocessing spectra were effective in improving the quality of calibration models with the best PLS calibration model selected for Savitzky-Golay smoothing spectra which had the lowest RMSECV (0.039) and highest RPD_cal_ value (4.64). Using this PLS model, a prediction for quantification of luwak content was calculated and resulted in satisfactory prediction performance with high both RPD_*p*_ and RER values.

## 1. Introduction

Coffee is one of the most important food commodities worldwide. Among all commodity traded in the world, coffee is number two after crude oil [1]. There are two important species of coffee which has economic significance in the global coffee trade, species* arabica* (*Coffea arabica*) and* robusta* (*Coffea canephora*). Another important type of coffee is luwak coffee or Asian palm civet coffee or kopi luwak (Indonesian words for coffee and palm civet) which is well known as the world's priciest and rarest coffee [2].

Luwak coffee is any coffee bean (arabica or robusta) which has been eaten and passed through the digestive tract of Asian palm civet (*Paradoxurus hermaphroditus*), which uses its keen senses to select only the best and ripest berries. As a result, its rarity as well as the coffee's exotic and unique production process ultimately accounts for its high selling price, approximately a hundred times higher than regular coffee (International Coffee Organization, http://www.ico.org/prices/pr-prices.pdf).

As one of the most profitable trading products, luwak coffee has been a target for fraud trading by mixing luwak coffee with other cheaper coffee. In order to protect the authenticity of luwak coffee and protect consumer from luwak coffee adulteration, it is very important to develop a robust and easy method for adulteration detection and quantification in luwak coffee. Recently, food authentication is a major challenge that has become increasingly important due to the drive to guarantee the actual origin of a product and for determining whether it has been adulterated with contaminants or filled out with cheaper ingredients [3].

At present, there is no internationally accepted method of verifying whether a roasted bean is luwak coffee or non-luwak coffee. Traditionally, coffee aroma has been used to characterize coffee quality. Sensory panel evaluation is commonly used to assess the aroma profile of coffee. However, this technique has some limitations. For example, it is quite difficult to train the panel effectively in order to limit subjectivity of human response to odors and the variability between individuals [4]. Jumhawan et al. [5] used gas chromatography coupled with quadruple mass-spectrometry (GC-Q/MS) to discriminate luwak and regular coffee which resulted in high coefficient of determination (*R*^2^) = 0.965. However, this method is quite expensive analysis with chemical waste included. Indonesia as one of the most important players in luwak coffees production is now just starting to develop an advanced technology for coffee processing. It is including a search for a novel inspection system for luwak coffees characterization. This technology is very important for coffee industry to protect high expensive luwak coffees from any adulteration.

In the previous study, Souto et al. [6] reported the use of UV-Visible spectroscopy as a simple analytical method for the identification of adulterations in ground roasted coffees (due to the presence of husks and sticks). This UV-Vis based analytical method is one of the most common and inexpensive techniques used in routine analysis and it will be compatible with situation in Indonesia for further technology development. Therefore, in this research, we attempt to use UV-Visible spectra combined with chemometrics methods (PLSR/partial least squares regression) to establish a rapid and simple method for quantification of adulteration in luwak-arabica coffee blend.

## 2. Materials and Methods

### 2.1. Sample Preparation

An amount of 1 kg ground roasted luwak robusta coffee (Indonesian wild palm civet coffee) was collected directly from coffee farmers at Liwa, Lampung, Indonesia (Hasti coffee Lampung). Another 1 kg ground roasted arabica coffee was also provided for making luwak coffee adulteration. All coffees were roasted in a home coffee roaster (Feike Roaster, W3000) at temperature of 210°C for 15 minutes (medium roasting). All coffees were grinded using home coffee grinder. Since particle sizes in coffee powder have significant influence on spectral analysis, it is important to use the same particle size in coffee powder samples [7]. In this research we use particle size of 420 *μ*m by sieving through a nest of US standard sieves (mesh number of 40) on a Meinzer II sieve shaker (CSC Scientific Company, Inc., USA) for 10 minutes. The experiments were performed at room temperature (around 27–29°C). In this research, we prepared 98 samples of coffee samples which consist of two types of samples, unadulterated (49 samples) and adulterated samples (49 samples). Unadulterated samples consist of 100% luwak coffee only and adulterated samples consist of luwak coffee with adulteration (adulterated with arabica coffee in the level of adulteration 10% (10 samples), 20% (10 samples), 30% (10 samples), 40% (10 samples), and 50% (9 samples)).

For developing and evaluating calibration model, the samples were divided into two groups: calibration and prediction sample set, respectively. Calibration sample set has 58 samples (24 unadulterated and 34 adulterated samples) and it is going to be used for developing calibration model with full cross-validation method. Prediction sample set has 40 samples (25 unadulterated and 15 adulterated samples) and this set is going to be used for evaluating the performance of developed calibration model.  Table 1 shows the detailed information on the samples used in this study.

An aqueous extraction procedure of the coffee samples was performed as described by Souto et al. [6] and Yulia and Suhandy [8]. First, 1.0 g of each sample was weighed and placed in a glass beaker. Then, 10 mL of distilled water was added at 90–98°C and then mixed with magnetic stirring (Cimarec™ Stirrers, model S130810-33, Barnstead International, USA) at 350 rpm for 5 min. Then the samples were filtered using a 25 mm pore-sized quantitative filter paper coupled with an Erlenmeyer. After cooling process to room temperature (for 20 min), all extracts were then diluted in the proportion of 1 : 20 with distilled water. UV-Vis-NIR spectra from the aqueous extracts were acquired using a UV-Vis spectrometer (GENESYS™ 10S UV-Vis, Thermo Scientific, USA).

### 2.2. Instrumentation and Spectra Data Acquisition

UV-Vis spectra in the range of 190–700 nm were acquired by using a UV-Visible spectrometer (GENESYS 10S UV-Vis, Thermo Scientific, USA) equipped with a quartz cell with optical path of 10 mm and spectral resolution of 1 nm at 27–29°C. Before the measurement step, blank (the same distilled water used in extraction process) was placed inside of the sample cell to adjust the 100% transmittance signal.

### 2.3. Spectral Data Analysis

All recorded spectra data were transferred to computer via USB flash disk and then converted the spectra data from  .*csv* extension into an excel data (.*xls*). Spectral preprocessing is required to remove physical phenomena in the spectra and to remove any irrelevant information such as noise and scattering effect. Recently many preprocessing methods are available in the commercial chemometric analysis tools. Some preprocessing methods were applied, including smoothing (moving average, median filter, and Savitzky-Golay smoothing), multiplicative scatter correction (MSC), and standard normal variate (SNV). The averaging technique is used to reduce the number of wavelengths or to smooth the spectrum of coffee solutions. It is also used to optimize the signal-to-noise ratio [9]. The MSC and SNV are designed to reduce the (physical) variability between samples due to scatter and adjust for baseline shifts between samples [10]. The MSC and SNV have the capability to remove both additive and multiplicative effects in the spectra [11].

Principal component analysis (PCA) was performed before developing the calibration model to determine any relevant and interpretable structure in the data and to detect outliers through the analysis of the Hotelling's T^2^ and squared residuals statistics [12]. PCA searches for directions of maximum variability in sample grouping and uses them as new axes called principal components (PC) that can be used as new variables, instead of the original data, in further calculations [13]. PCA results showed that there were no outliers detected in calibration and prediction data sets.

Partial least squares (PLS) regression was used to develop the calibration model for original and preprocessing spectra. PLS finds the directions of greatest variability by considering not only spectral data but also luwak content data, with new axes, called PLS factors (F) or latent variables [13]. The best number of latent variables (LVs) is then chosen according to a commitment between the lowest root mean square error of cross-validation (RMSECV) and the lowest number of latent variables [14, 15]. The quality of the calibration model was evaluated using the following statistical parameters: coefficient of determination between predicted and measured luwak content in luwak-arabica blend (*R*^2^), root mean square error of calibration (RMSEC), root mean square error of cross-validation (RMSECV), bias between actual and predicted luwak content, and ratio prediction to deviation (RPD) value (RPD_cal_ = SD_validation set_/RMSECV) [16]. A value of *R*^2^ indicates the percentage of the variance in the *Y* variable (luwak content in luwak-arabica blend) that is accounted for by the *X* variable (spectral data). As mentioned by Saeys et al. [17], a calibration model with *R*^2^ value greater than 0.91 is considered to be an excellent calibration, while an *R*^2^ value between 0.82 and 0.90 results in good prediction [18, 19]. A small difference between RMSEC and RMSECV value was also important to avoid “overfitting” in the calibration model [20]. The calibration model should have as high as possible RPD value. The RPD value is desired to be larger than 3 for an acceptable calibration [21]. Calibrations with RPD values between 1.4 and 2 indicate a satisfactory performance of the model which can be useful for rapid screening of samples and may be improved using different sampling strategies or modelling methods and <1.4 indicated an unacceptable model [22].

Spectra preprocessing, PCA, and PLS regression were performed using The Unscrambler® version 9.8 (CAMO, Oslo, Norway), a statistical software for multivariate analysis. A student's paired *t*-test was performed using Statistical Package for the Social Science (SPSS) version 11.0 for Windows in order to evaluate the significance level of the developed model.

## 3. Results and Discussions

### 3.1. UV-Visible Spectra of Coffee Solution Samples in Range 190–700 nm

**Figure 1:**
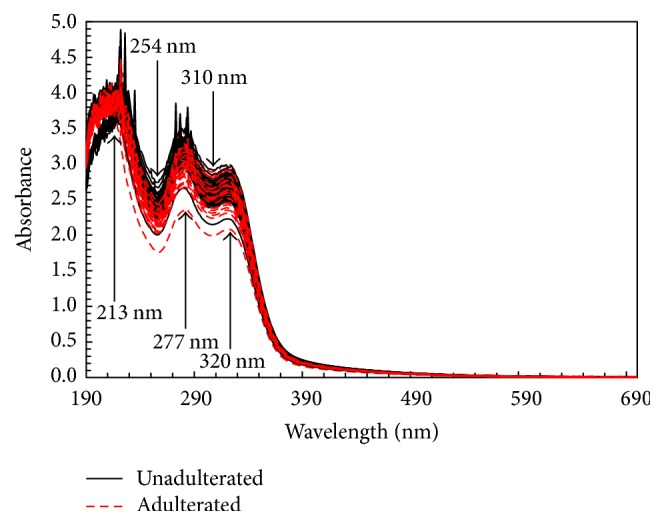
Original spectra of unadulterated and adulterated coffee samples in the UV-Vis region.

Figure 1 shows the original spectra of 98 coffee solution samples in range 190–700 nm. Several peaks can be observed at 213, 277, and 320 nm. It can be seen that all the spectra have similarity in spectral shape and absorbance. The spectra of unadulterated (solid line with black color) and adulterated samples (dashed line with red color) overlap, and it is difficult to detect obvious division between them. The high noise was also observed. Thus, it is necessary to apply appropriate multivariate analysis methods to extract useful information from the spectra, minimize the noise, and build calibration models for quantification of luwak content in luwak-arabica coffee blend.

### 3.2. Developing PLS Calibration Model for Quantification Luwak Content

**Figure 2:**
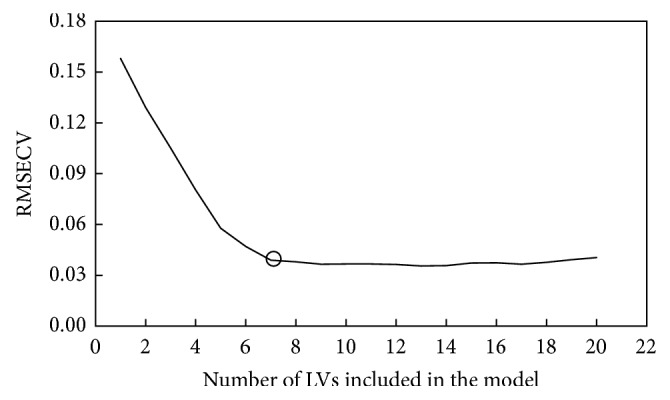
Number of LVs versus RMSECV for PLS calibration model for determination of luwak content.

Using the PLS regression method the calibration and validation were performed for original and preprocessing spectra (Table 2). The calibration model with the original spectra resulted in a high coefficient of determination (*R*_cal_^2^ = 0.97). In terms of RMSECV, all the preprocessing of spectra (except for mean centering) was effective in improving the quality of calibration model. For smoothing spectra, the calibration model was improved by moving average, media filter, and Savitzky-Golay (SG). Using MSC and SNV spectra, the PLS calibration model was significantly improved as the RMSECVs were decreased. The best PLS calibration model was selected for SG smoothing spectra with window width of 13 points (6-1-6) and polynomial order = 2 which had the lowest RMSECV (0.039) and highest RPD_cal_ value (4.64). This calibration model has 7 optimal numbers of LVs as indicated in Figure 2.

**Figure 3:**
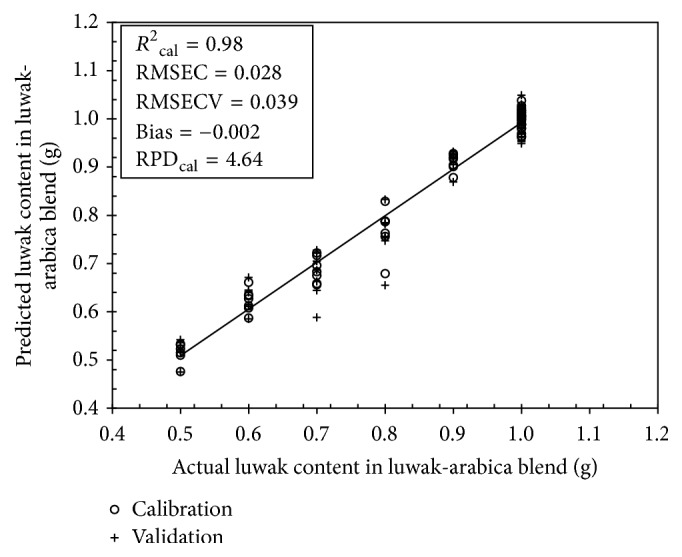
PLS calibration and validation model for luwak content determination using SG smoothing spectra in the range 190–700 nm.

This PLS calibration model was comparable to that reported by Wang et al. [23] for Kona coffee content determination in several brands of commercial Kona coffee blend with *R*^2^ = 0.996 for ground Kona coffee blends and *R*^2^ value of 0.999 for brewed Kona coffee. Using metabolomics approach and orthogonal projection to latent structures (OPLS) prediction technique, Jumhawan et al. [24] developed two prediction OPL models to quantify the degree of coffee adulteration for certified and commercial luwak coffee with *R*^2^ = 0.975 and *R*^2^ = 0.987, respectively. The scatter plot between actual and predicted luwak content in the best PLS calibration model using SG smoothing spectra is presented in Figure 3.

### 3.3. Prediction Result Using the Best PLS Calibration Model

To evaluate the performance of the best PLS calibration model, the independent prediction sample set (the sample used for prediction is different with sample used for developing calibration model) is projected onto the best PLS calibration model yielding the prediction set results. From this projection, the root mean square error of prediction (RMSEP), the coefficient of prediction (*R*^2^_*p*_), the range error ratio (RER) (RER = (maximum − minimum)_reference value_/RMSEP) [25], and the RPD for prediction (RPD_*p*_ = SD_prediction set_/RMSEP) were obtained. Both RPD_*p*_ and RER are good indicators of evaluating model performance [26, 27]. As for guidance, when RPD_*p*_ is greater than 3 and RER is greater than 10 the calibration model is considered to be successful [28–30].

**Figure 4:**
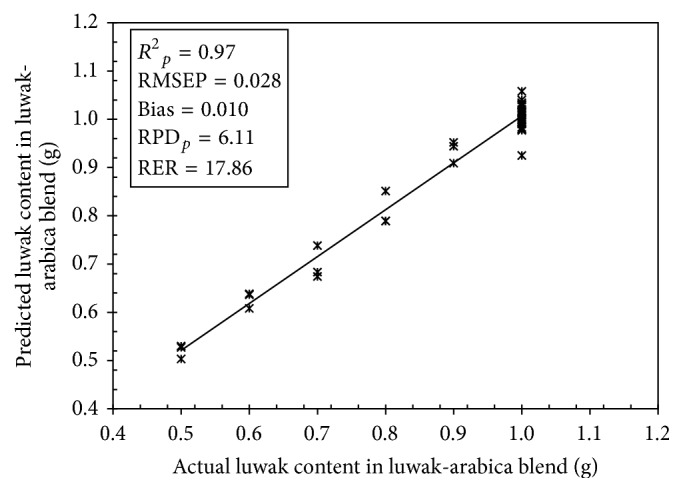
Scatter plot of actual versus predicted luwak content calculated using the best PLS calibration model.

Figure 4 shows the results for luwak content determination based on the best PLS calibration model with SG smoothing spectra. It has high *R*^2^_*p*_ = 0.97 with low RMSEP = 0.028. From the RPD_*p*_ value, it can be seen that the RMSEP was much lower than the standard deviation (SD = 0.171) of reference data which resulted in high RPD_*p*_ value. The obtained RER is also quite good (17.86). By a 95% confidence paired *t*-test there were no significant differences between actual and predicted luwak content. This indicates that an accurate calibration model can be developed for the determination of luwak content in luwak-arabica coffee blends using UV-Vis spectroscopy and PLS regression.

### 3.4. Selection of Important Wavelengths

**Figure 5:**
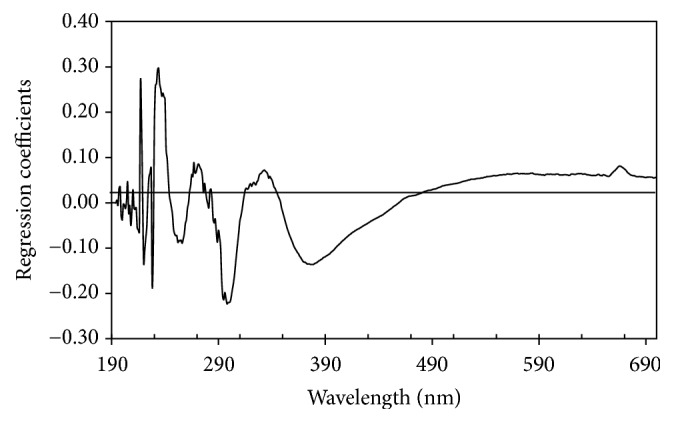
Regression coefficients versus wavelength of coffee samples.

**Figure 6:**
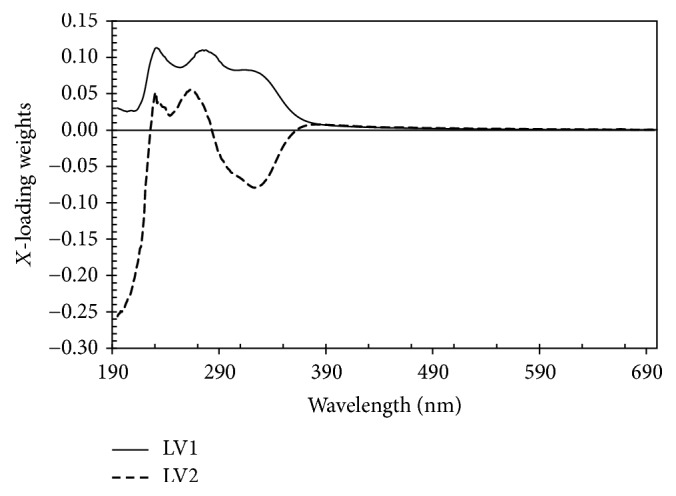
*X*-loading weights versus wavelength of the top two latent variables (LV1 and LV2) of coffee samples.

In order to understand the complexity of developed PLS model, regression coefficients and *x*-loading weights of the best PLS model were presented in Figures 5 and 6, respectively. The *x*-loadings show how well the *x*-variable (wavelengths) is taken into account by the model components. It can be used to understand how much each *x*-variable (wavelengths) contributes to the meaningful variety variation in the data and to interpret variable relationships. It is also useful to interpret the meaning of each model component. The loading weights show how much each wavelength (*x*-variables) contributes to explaining the response variation (degree of adulteration) along each model component. The loading weights are normalized, so that their lengths could be interpreted as well as their directions. Wavelengths (*x*-variables) with large loading weight values are important for the determination of luwak content in luwak-arabica blend. With a similar function, regression coefficients are primarily used to check the effects of different wavelengths (*x*-variables) in determination of luwak content in luwak-arabica blend. Large absolute values indicate the importance and significance of the effects of the wavelengths. According to Figure 5 we can see several peaks and valleys at certain wavelengths which were considered to be more important for determination of luwak content in luwak-arabica blend, such as 228, 256, 274, 299, 332, and 376 nm. In Figure 6 we can notice several wavelengths with high contribution to the developed PLS model at 228, 246, 274, and 320 nm. We can see that all important wavelengths are in the ultraviolet spectral region. In the visible region we could not find any important wavelengths indicated with low *x*-loading and regression coefficients in the visible region. It is shown that the determination of luwak content in luwak-arabica blend is mainly characterized in the ultraviolet region. The several observed important wavelengths in this study are closely related to the absorbance of several important chemical compositions in roasting coffee. For example, the wavelength at 276 nm can be found both in *x*-loading and in regression coefficient plot and this wavelength is related to the absorbance of caffeine while wavelength at 320 nm is related to absorbance of caffeic acid [6]. The wavelengths at 246, 299, and 320 nm are closely related to the absorbance of chlorogenic acids (CGA) [31]. The wavelength at 256 nm is closely related to the absorbance of vanillic acid.

## 4. Conclusion

In this research, the determination of luwak content in luwak-arabica coffee blends was achieved by using UV-Visible spectroscopy and PLS regression. The best PLS calibration model with Savitzky-Golay smoothing spectra resulted in satisfactory prediction with excellent value both for RPD and for RER. Several wavelengths with high contribution to the luwak content determination were confirmed including 276 nm which is related to the absorbance of caffeine while wavelength at 320 nm is related to absorbance of caffeic acid. This research shows the possibility of developing a simple, rapid, and economic method for determining luwak content in luwak-arabica coffee blends using UV-Visible spectroscopy and multivariate analysis.

## Figures and Tables

**Table 1:** Descriptive statistic of luwak content in coffee samples used for developing calibration and prediction in luwak-arabica coffee blend.

Item	Calibration and validation sample set	Prediction sample set
Number of samples	58	40
Range	1.0~0.5	1.0~0.5
Mean	0.828	0.888
Standard deviation	0.181	0.171
Unit	Gram	Gram

**Table 2:** Calibration and validation results for determination of luwak content in luwak-arabica blend using original and preprocessing spectra in the range 200–450 nm.

Type of spectra	*F*	*R*_cal_^2^	RMSEC	RMSECV	Bias	RPD_cal_
Original	7	0.97	0.029	0.062	−0.003	2.92
Moving average smoothing with 5 segments	7	0.97	0.029	0.042	−0.002	4.31
*Savitzky-Golay smoothing with window width of 13 points and polynomial order 2*	*7*	*0.98*	*0.028*	*0.039*	*−0.002*	*4.64*
Median filter smoothing with 3 segments	7	0.98	0.027	0.050	−0.003	3.62
MSC	7	0.98	0.026	0.055	−0.001	3.29
SNV	7	0.98	0.025	0.054	−0.001	3.35
Baseline offset	7	0.98	0.027	0.060	−0.002	3.02
Mean centering	7	0.97	0.029	0.062	−0.003	2.92
